# Cross-clade simultaneous HIV drug resistance genotyping for reverse transcriptase, protease, and integrase inhibitor mutations by Illumina MiSeq

**DOI:** 10.1186/s12977-014-0122-8

**Published:** 2014-12-23

**Authors:** Dawn M Dudley, Adam L Bailey, Shruti H Mehta, Austin L Hughes, Gregory D Kirk, Ryan P Westergaard, David H O’Connor

**Affiliations:** Department of Pathology and Laboratory Medicine, University of Wisconsin School of Medicine and Public Health, Madison, WI USA; Department of Epidemiology, Epidemiology and Oncology, Johns Hopkins University, Baltimore, MD USA; Department of Biology, University of South Carolina, Columbia, South Carolina USA; Departments of Medicine, Epidemiology and Oncology, Johns Hopkins University, Baltimore, MD USA; Department of Medicine, Division of Infectious Disease, University of Wisconsin School of Medicine and Public Health, Madison, WI USA

**Keywords:** Human immunodeficiency virus, Antiretroviral therapy, HIV drug resistance, Drug resistance genotyping, Illumina MiSeq, Integrase inhibitor genotyping, Deep sequencing

## Abstract

**Background:**

Viral resistance to antiretroviral therapy threatens our best methods to control and prevent HIV infection. Current drug resistance genotyping methods are costly, optimized for subtype B virus, and primarily detect resistance mutations to protease and reverse transcriptase inhibitors. With the increasing use of integrase inhibitors in first-line therapies, monitoring for integrase inhibitor drug resistance mutations is a priority. We designed a universal primer pair to PCR amplify all major group M HIV-1 viruses for genotyping using Illumina MiSeq to simultaneously detect drug resistance mutations associated with protease, nucleoside reverse transcriptase, non-nucleoside reverse transcriptase, and integrase inhibitors.

**Results:**

A universal primer pair targeting the HIV *pol* gene was used to successfully PCR amplify HIV isolates representing subtypes A, B, C, D, CRF01_AE and CRF02_AG. The universal primers were then tested on 62 samples from a US cohort of injection drug users failing treatment after release from prison. 94% of the samples were successfully genotyped for known drug resistance mutations in the protease, reverse transcriptase and integrase gene products. Control experiments demonstrate that mutations present at ≥ 2% frequency are reliably detected and above the threshold of error for this method. New drug resistance mutations not found in the baseline sample were identified in 54% of the patient samples after treatment failure. 86% of patients with major drug resistance mutations had 1 or more mutations associated with drug resistance to the treatment regimen at the time point of treatment failure. 59% of the emerging mutations were found at frequencies between 2% and 20% of the total sequences generated, below the estimated limit of detection of current FDA-approved genotyping techniques. Primary plasma samples with viral loads as low as 799 copies/ml were successfully genotyped using this method.

**Conclusions:**

Here we present an Illumina MiSeq-based HIV drug resistance genotyping assay. Our data suggests that this universal assay works across all major group M HIV-1 subtypes and identifies all drug resistance mutations in the *pol* gene known to confer resistance to protease, reverse transcriptase and integrase inhibitors. This high-throughput and sensitive assay could significantly improve access to drug resistance genotyping worldwide.

**Electronic supplementary material:**

The online version of this article (doi:10.1186/s12977-014-0122-8) contains supplementary material, which is available to authorized users.

## Background

Antiretroviral therapy has been shown to be the most potent intervention for both treating and preventing HIV [1,2]. However, the effectiveness of antiretroviral drugs is threatened by the development and transmission of drug resistant virus [3,4]. HIV drug resistance genotyping is a clinically important tool to detect the emergence of viral resistance and maximize the benefit of current treatment options [5,6]. New sequencing techniques can improve sensitivity of drug resistance genotyping while also reducing costs. We recently published a paper outlining the benefits of using Roche/454 pyrosequencing to detect HIV drug resistance mutations with a capacity to reduce costs by 5-fold [7]. As sequencing technologies have continually improved, the genotyping methods used in clinical settings to detect drug resistance have failed to evolve at a comparable pace. Here we present an updated deep sequencing genotyping assay utilizing the Illumina MiSeq. Advantages of this novel method over our Roche/454 approach include fewer systemic basecalling sequencing errors, increased multiplexing, use of a FDA-approved sequencing platform, detection of drug resistance to integrase inhibitors in addition to reverse transcription inhibitors and protease inhibitors with a single amplicon, and finally, simultaneous genotyping of all major group M HIV subtypes with a single set of amplification primers.

Detection of Integrase inhibitor drug resistance is particularly noteable because integrase inhibitors are now recommended by the department of Health and Human Services as part of first-line antiretroviral therapy in the United States [8]. The use of integrase inhibitors as part of initial antiretroviral therapy is expected to increase because of the recent FDA approval of a new once daily integrase inhibitor (elvitegravir) that can be co-formulated with emtricitabine, tenofovir disoproxyl fumarate and cobicistat as a single pill once daily regimen (STRIBILD) [9]. Current genotyping methods used to detect drug resistance to integrase inhibitors are limited and typically involve a separate assay from that used to identify drug resistance to reverse transcriptase and protease inhibitors [10,11]. However, due to proximity of the integrase protein-coding region to the reverse transcriptase and protease protein-coding region in the viral genome, it is feasible to incorporate drug resistance genotyping of all three gene products together from a single PCR amplicon. By using a single amplicon, no additional labor or expense is added to the assay already detecting protease and reverse transcriptase drug resistance mutations.

The FDA-approved HIV drug resistance genotyping assays currently in use were developed using HIV group M subtype B viruses [12]. However, subtype C comprises about half of the worldwide epidemic followed by subtype A (12%) and then B (11%) [13]. Current commercial genotyping assays perform rather poorly with non-subtype B viruses because seven sets of sequencing primers must bind to highly variable targets during sequencing [12,14]. Therefore, non-B assays require more replication and for many samples, fail altogether with the current commercially available genotyping assays. There have been many successful adaptations of the commercial Sanger-based genotyping assay to improve genotyping of non-B HIV subtypes, however, these assays do not include detection of integrase inhibitor mutations and are less sensitive than next-generation sequencing approaches [15-21].

Cost is a major barrier to drug resistance genotyping in resource-limited settings. Deep sequencing technologies have significantly reduced the cost of sequencing but are not yet incorporated into standard HIV drug resistance genotyping. An Illumina MiSeq sequencer can yield over 25 million sequencing reads per run with the latest technology (MiSeq reagent kit v3). Through multiplexing, 96 samples can be processed in one run, bringing the cost of sequencing alone to < $10/sample. This is 3-fold less than Sanger-based sequencing. In addition, the clonal nature of deep sequencing methods along with the large number of sequencing reads generated improves sensitivity for the detection of mutations compared to Sanger-based sequencing [12,22,23]. Lastly, the Illumina MiSeq platform is currently the least error-prone deep sequencing method [24,25].

## Results and discussion

### PCR primers amplify major group M HIV subtypes A, B, C, D, CRF01_AE and CRF02_AG

HIV sequences from all subtypes found in the Los Alamos National Labs HIV database (http://www.hiv.lanl.gov) were used to design a universal primer set to amplify all major HIV subtypes as described in the Methods section (HIV2252-F and HIV5073-R). We obtained HIV isolates from the International Panel of 60 HIV-1 isolates from the NIH AIDS Research and Reference Reagent Program, Division of AIDS, NIAID, NIH (cat. #11412) representing subtypes A, B, C, D, CRF01_AE and CRF02_AG. We selected 19 isolates to represent geographically diverse regions as shown in Table 1, isolated viral RNA and performed one step RT-PCR to create PCR amplicons according to the protocol outlined in the Methods section. Despite a wide range of TCID50 and/or P24 content in the panel isolates tested, all isolates yielded a strong amplification product after 40 cycles of PCR as visualized on a 1% agarose gel (Additional file 1). These results suggest that the primer pair designed for this method could be used to amplify all major group M HIV subtypes.

**Table 1:** **HIV-1 subtypes tested with the universal primer set**

**Virus**	**Subtype**	TCID/mL*	P24 ng/mL*	**RT-PCR**
92UG_029	A	1.26x10^5^	150	**+**
93RW_024	A	NA	NA	**+**
00KE-KER2008	A	10^2.60^	57	**+**
84US_MNp	B	1.02x10^4^	634	**+**
85US_BA-L	B	5.62x10^6^	39.3	**+**
91US_1	B	10^5.0^	294	**+**
94US_33931N	B	10^3.10^	235	**+**
00TZ_A246	C	1.45x10^4^	NA	**+**
02ET_14	C	10^4.10^	12.2	**+**
94IN_20635-4	C	NA	NA	**+**
90SE_364	C	10^6.0^	157	**+**
98US_MSC5016	C	10^3.85^	11.5	**+**
93UG_065	D	NA	NA	**+**
98UG_57128	D	10^2.39^	13	**+**
99UG_A03349M1	D	2.05x10^4^	290	**+**
90TH_CM244	CRF01_AE	4.1x10^4^	88	**+**
97TH_NP1525	CRF01_AE	7.11x10^3^	90	**+**
91DJ_263	CRF02_AG	2.3x10^4^	NA	**+**
98US_MSC5007	CRF02_AG	4x10^4^	127	**+**

* Provided by the NIH AIDS Research and Reference Reagent Program.

### Error associated with the genotyping assay

**Figure 1:**
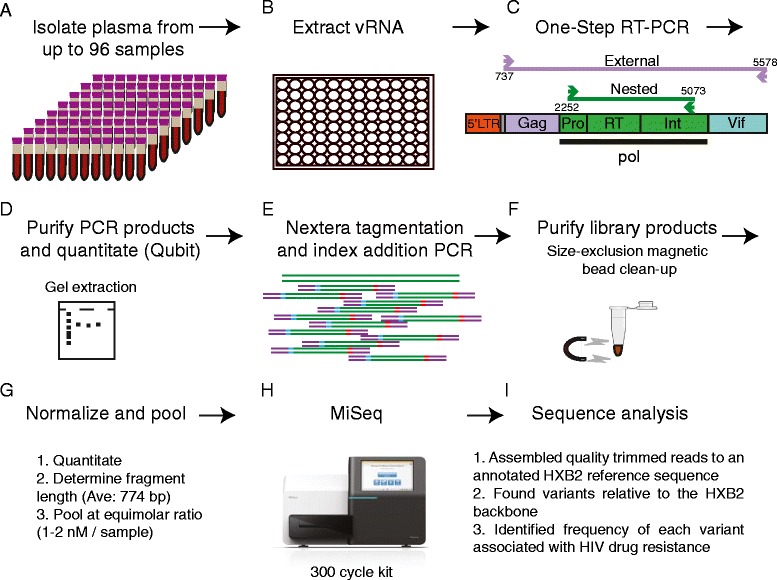
**Preparation and sequencing of samples. (A)** Plasma is isolated from whole blood from up to 96 samples. **(B)** Viral RNA is isolated from up to 1 ml of plasma. **(C)** Viral RNA is used in a one-step RT-PCR amplification of a 2.8 kb region of the *pol* gene. When nested PCR is required, a 4.8 kb region is amplified as an external PCR followed by the 2.8 kb nested PCR of the *pol* gene. **(D)** PCR products are purified either by gel electrophoresis followed by gel extraction or through size-exclusion magnetic beads and then quantitated using the Qubit system. **(E)** Purified products are randomly fragmented and subjected to a limited cycle PCR to add sequencing adaptors and indices used for multiplexing samples. **(F)** Newly created libraries are purified by size-exclusion magnetic beads to remove short fragments. **(G)** The average size of the library fragments are calculated by bioanalysis and final concentration of the libraries calculated by Qubit are used to normalize each library and pool multiple libraries together at equimolar ratios. **(H)** Libraries are sequenced on the Illumina MiSeq. **(I)** Geneious Pro Software is used to trim sequencing reads based on quality scores and assemble the reads to a HXB2 reference sequence annotated with HIV drug resistance mutations. Geneious is used to identify variants within each sample relative to HXB2. Finally, variants associated with drug resistance mutations were extracted and their frequencies noted. Details about the analysis parameters are outlined in the Methods section.

To assess the error associated with the RT-PCR amplification and sequencing of samples, a clonal virus stock was generated from a plasmid containing the HXB2 HIV sequence. We PCR amplified and sequenced the stock as described in the Methods section and as depicted in Figure 1. We assessed all nucleotide variants present above a 0.1% frequency across the entire Pol region sequenced from two independent amplifications and sequencing runs of the HXB2 clonal stock amplicons. Any variant found in our clonal virus stock is expected to be due to PCR or sequencing errors. The frequency of these variants determined our error limit threshold for this assay. Nucleotide polymorphisms that result in a non-synonymous amino acid changes are presented in Additional file 2. Most nucleotide variants were present at frequencies less than 0.4%, however, two regions contained variants at frequencies between 0.6% and 0.9%. One insertion was present at a frequency of 0.9% that was associated with the end of the first of two back-to-back long homopolymer runs (AAAAAAGAAAAAA) found near the K101 and K103 drug resistance mutations, but not within them. Another single nucleotide polymorphism was found at a 0.6-0.7% frequency downstream of the integrase drug resistance mutations (nucleotide position 2779 (R275R in integrase)). This polymorphism was a synonymous change that did not alter the amino acid sequence. These results suggest that we see some evidence of increased mutations associated with homopolymers in Illumina data, as has been published, but that those mutations are not present above a 1% frequency nor do they impact the codons directly associated with drug resistance [26]. In addition, errors associated with the GGC sequence motif were also not found above a frequency of 0.5%. We also detected five variants present at 99.9-100% frequencies that are likely differences between the modified HXB2 plasmid we sequenced and the published HXB2 strain and were ignored as variants caused by PCR or sequencing errors (non-synonymous changes shown in Additional file 2). Overall, no variants were found above a 0.9% frequency in our clonal HIV stock.

When specifically assessing nucleotide variants associated with amino acid changes linked to HIV drug resistance across the *pol* gene, we found that all variants within drug resistance sites were present at frequencies below 0.3% of the viruses sampled from our clonal stock (Additional file 2). Therefore, given both the error limits within the drug resistance sites and outside of the drug resistance sites, variants found at frequencies greater than 1%, are likely authentic, while those below this threshold may result from RT-PCR and sequencing artifacts. Repeated sequencing of a subset of samples revealed that variants above 2.0% are consistently detected, while some variants found at frequencies between 1.0% and 2.0% are not (Additional file 2). Therefore, we designated 2.0% as the minimum threshold frequency for variants in subsequent experiments. Previous studies suggest that incorrect nucleotide incorporation associated with PCR error typically occurs at rates under 2.0%, supporting our finding that variants found at a greater than 2.0% frequency are likely true variants [27]. Also, the error rates described here are very specific to the precise protocol, polymerases and sequencing kit used in this manuscript. Error rates should be reassessed if any changes to the method are made.

To determine whether nested PCR increased the error associated with low-frequency mutations, we performed the same test as described above, but subjected the clonal virus stock to two rounds of PCR under the same conditions used for primary samples. Once again, variants from the expected HXB2 sequence were present below a frequency of 1.0% (Additional file 2).

We previously published a high throughput next-generation-based HIV drug resistance genotyping technique based on the Roche/454 sequencing platform [7]. One of the disadvantages of this system was the error associated with nucleotide homopolymer runs resulting from the sequencing chemistry used in the Roche/454 system. Sequence regions with homopolymer runs of three or more nucleotides are often miscalled, presenting a problem for some important drug resistance mutations like K103N and K65R that are encoded by homopolymeric nucleotide sequences. The competitive addition of reversible terminators used in Illumina’s sequencing technology greatly reduces homopolymer errors resulting in more accurate identification of drug resistance mutations within these important sites. After trimming, the nucleotides representing the homopolymers in K103N and K65R mutations found in our patient samples maintained Phred quality scores > Q30 (or p = 0.001), lending greater confidence to the nucleotide bases called in these regions than was afforded by the Roche/454 assay where quality scores in these regions often dropped below Q20.

### PCR amplification using specimens from ALIVE

We obtained plasma samples collected from adults with a history of injection drug use who participated in the AIDS Linked to the IntraVenous Experience (ALIVE) study in Baltimore, MD [28]. Previous research with this cohort showed that virologic failure occurs with high frequency when participants experience incarceration, but it is not known whether the viral rebound that occurs in this setting is associated with development of drug resistance [29]. To answer this question while testing our method on primary HIV isolates, we genotyped HIV RNA from stored plasma specimens obtained from ALIVE participants. First, we isolated plasma viral RNA from 29 patients with samples from at least two time points for a total of 62 samples (see Additional file 3). 94% (58/62) of the samples were successfully amplified and sequenced, though 9 of these samples required nested PCR amplification. Most of the samples that either failed or required nested PCR either had low viral load values (<1,000 copies/ml) or were 5–13 years old and underwent multiple freeze-thaw cycles prior to PCR amplification (see Additional file 3). Of the samples that were successfully sequenced with viral loads above 2,000 copies/ml, 94% (48/51) of the samples amplified with one round of PCR using the universal primer set. Since each unique patient sample amplified in at least one time point and our primers are universal for multiple subtypes, we do not believe that the subtype of the patient virus was the reason for the failure to amplify in three samples. In any event, our sequences clustered with subtype B virus in phylogenetic analysis of the pol gene sequences (Additional file 4). Overall, we expect that if this method was used on recently collected samples with viral loads greater than 2,000 copies/ml for timely genotyping assays, nested PCR would not be required. Lastly, we were able to sequence primary isolate samples with a viral load as low as 799 copies/ml with nested PCR and 1070 copies/ml with a single round of PCR (see Additional file 3). However, each patient sample likely exhibits a unique limit of detection depending on the sequence concordance between the primers and patient virus.

### Sequencing using specimens from ALIVE

**Figure 2:**
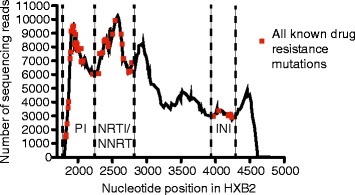
**Representative sequence coverage across the** ***pol*** **gene.** The number of sequences representing each nucleotide across the pol gene (i.e. “coverage”) is shown for all sites that differed from HXB2 in a representative sample (sample 16 v11). Sites of all known drug resistance mutations are over-layed as red squares and the graph is divided into sections representing mutations associated with protease, reverse transcriptase or integrase drug resistance for reference.

Including controls, 63 PCR amplicons were fragmented and pooled together in a single MiSeq run. The cluster density of the sequencing run was 663 k/mm^2^ and the average number of sequence reads obtained per sample was 146,780 (range: 74,848-244,428, stdev: 34,556). The coverage, or number of sequences that represent each drug resistance site after mapping to a reference sequence, varied between each patient and across the *pol* gene. Figure 2 shows the coverage of a representative sample (patient 16) with 142,169 total reads. All nucleotides present between the first protease inhibitor resistance mutation (HXB2 position 1826) and last integrase inhibitor drug resistance mutation (HXB2 position 4238) were represented by more than 1,000 sequence reads in this patient (red squares in Figure 2). The goal of this method to generate a depth of coverage of >1000 sequencing reads/nucleotide position and in fact, at the major drug resistance sites, the average coverage of each mutation was over 1000 sequence reads (Table 2). Based on previous next-generation sequencing work, to accurately detect mutations at a frequency of 1.0%, minimal coverage requirements have ranged from 300 to 1850 sequencing reads [22,30-32]. With our minimum frequency threshold set to 2.0%, requiring a minimum of 1000 sequencing reads is conservative relative to previous publications. Across all samples, the average number of sequences representing each drug resistance site was 3,795 sequences. While coverage varied across the *pol* gene, the quality of all assembled sequences was very high due to the stringent trimming parameters (see Methods section) used during analysis.to remove nucleotides whose base call was deemed less than 99.9% accurate by the sequencing software prior to assembly (phred quality score > Q30 or p > 0.001). Note that this data was generated prior to the release of the latest MiSeq sequencing kits, which now have an average output capacity of 25 million reads per sequencing run. This would more than double the reads obtained per sample as well as double the sequence read coverage at each nucleotide site given the same pooling strategy.

**Table 2:** **Average sequencing read coverage for all drug resistance mutations identified in the ALIVE cohort**

**Gene product**	Drug resistance mutation*	**Ave. sequencing read coverage**
PR	L10IFVC	297
	V11I	294
	G16E	1022
	K20RMITV	1917
	**L24I**	**1039**
	**D30N**	**2169**
	**L33FIV**	**3140**
	M36LIV	3639
	**M46I/L**	**2609**
	**I50L/V**	**3895**
	F53LY	1213
	**I54VTALM**	**2122**
	D60E	3065
	I62V	3198
	L63P	3346
	I64LMV	2635
	A71VITL	3800
	G73CSTA	3045
	**V82ATFSL**	**3300**
	L89VIM	4199
	**L90M**	**4982**
	I93LM	5850
RT	**M41L**	**5014**
	A62V	4941
	**K70R**	**4324**
	V75I	10460
	V77I	4787
	V90I	4177
	**K103N**	**6048**
	**V106A/M/I**	**6492**
	V108I	7424
	**E138KAGQR**	**7401**
	**M184V/I**	**5906**
	**Y188LHC**	**5283**
	**G190ASE**	**4708**
	P225H	5764
	**M230LI**	**8668**
IN	**S147G**	**3123**

*Boldface mutations are major drug resistance mutations, non-boldface mutations are accessory mutations.

In general, across all patient samples and as exemplified in Figure 2, the coverage of the nucleotides associated with the protease inhibitor mutations found closest to the 5′ end of protease were lower than the number of sequences representing all other drug resistance mutation sites downstream. There are four accessory mutations (L10, V11, G16E and K20) and zero major mutations that sit close to the end of the original *pol* gene PCR amplicon that are sometimes affected by this reduced coverage (Table 2). These four sites are less represented because transposon-based fragmentation creates fewer fragments at the ends of the input DNA strand (original full-length *pol* gene amplicon) that have the necessary adaptor required for sequencing. Instead of 1000s of reads, sometimes these mutations are represented by hundreds of reads in this cohort (see Table 2). For the two mutations (L10 and V11) in our cohort that are represented by an average of 300 sequencing reads, a minimum frequency requirement could be increased to 6.5% to ensure that at least 20 reads are found containing the mutation, keeping the requirement the same as a mutation found at a 2% frequency in 1000 sequencing reads. This caveat to this approach could be solved by generating a primer that sits further upstream in the protease region, however, primers design is constrained when trying to keep the primers universal across multiple HIV clades. As mentioned above, with the latest MiSeq sequencing kits, coverage will also double at these sites. Lastly, reducing the multiplexing would also yield greater coverage per sample at this site if coverage of these mutations was a concern. The data from this patient cohort shows that this method combining 63 samples together results in characterization of major drug resistance mutations with thousands of sequencing reads representing most mutations. Based on our average coverage of 3,795 sequences, extrapolation would suggest that 96 samples could be pooled together and average coverage of major drug resistance mutations would still be over 1,000 sequences per drug resistance site when optimal cluster density is reached on the MiSeq sequencing chip. This would further increase by at least 2-fold by using the latest MiSeq reagent kit V3 technology.

Although transposon-based fragmentation is theoretically unbiased, we observed consistently greater coverage in the reverse transcriptase protein coding region than protease and integrase coding regions across all samples, as exemplified in Figure 2. This similar coverage pattern between different samples over the same genetic regions is similar to that previously observed with whole genome HIV and SIV sequencing using the same fragmentation method [33]. Therefore, it is likely that there is some bias in fragmentation that yields ideally sized fragments for sequencing in some parts of the HIV genome over others.

Lastly, the assembled sequences were aligned and a maximum likelihood phylogenetic analysis was performed along with reference sequences from the panel of HIV isolates that we PCR amplified with our universal primer set (Additional file 4). As shown in Additional file 4, all samples from this cohort cluster with subtype B viruses, including the HXB2 control samples that we sequenced. In addition, the two time points from each sample clustered with each other and not with other samples, indicating that there was no cross-contamination resulting in the changes in drug resistance mutations found between the two time points from each patient sample.

### Drug resistance mutations detected in specimens from ALIVE

**Figure 3:**
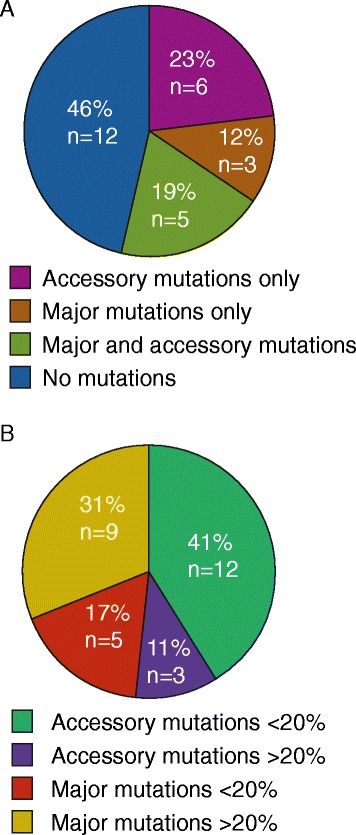
**Characterization of the drug resistance mutations identified in the ALIVE cohort after treatment failure. (A)** The frequency of different types of HIV drug resistance mutations found in 26 patient samples with after treatment failure. Major mutations are as defined by the Stanford drug resistance database and accessory mutations are all other mutations associated with drug resistance as designated by the International AIDS Society (IAS). **(B)** Percentages of the 29 emerging mutations found at the second time point but not first time for all participant samples that were major or accessory mutations and whether the mutations rose above the 20% threshold expected to be detected by commercial HIV drug resistance genotyping techniques.

To detect HIV drug resistance mutations in virus from the 29 ALIVE participants failing treatment after release from prison/jail, we analyzed the *pol* gene sequencing reads from two time points. First, the ALIVE participants successfully suppressed HIV before incarceration by using antiretroviral therapy. The first time point we sampled was at the time point just prior to this viral suppression. Some patients were drug naïve at this time point, but others were previously drug experienced (as outlined in Additional file 3) and either changed regimens or began taking ARTs after a break in treatment to reach viral suppression at the subsequent time point. The second time point represents the virus after treatment failure, as detected at an ALIVE study visit, after release from jail or prison. We obtained sequence data from both time points from 26/29 specimens. Relative to the first time point sequenced (baseline), new drug resistance mutations emerged in 54% (14/26) of the patients exhibiting treatment failure that were not detected at baseline (Figure 3A and Table 3). One additional sample (patient 27) contained major drug resistance mutations at both time points tested that may have contributed to treatment failure at the second time point (Table 4). These mutations, I54V, L90M, K103N and M184V, were present at frequencies >85% at both time points with most being present at >99% of sequences. The remaining samples without emerging drug resistance mutations contained primarily accessory mutations at baseline or at the second time point or very low level major drug resistance mutations that disappeared by the second time point and therefore likely do not contribute to treatment failure. Therefore, these patients may be failing treatment for reasons other than drug resistance mutations in the pol gene product. For example, some patients may fail due to lack of adherence or presence of mutations in Gag or the cytoplasmic tail of Env, which have been recently reported to affect drug resistance to protease inhibitors [34-36]. In the future it will be possible to expand our technique to include these regions outside of the *pol* gene, however, it would require amplification of more than one amplicon prior to fragmentation and sequencing.

**Table 3:** **Drug resistance mutations emerging after incarceration in the ALIVE cohort**

**Drug class**	**Mutation**	**Sample #(s) (% frequency)**
**Protease inhibitors**	L10IFVC	19 (7.9%)
	V11I	25 (6.0%)
	G16E	25 (3.2%)
	K20RMITV	24 (7.0%)
	M36LIV	24 (5.5%)
	**M46IL**	27 (7.3%)
	F53LY	16 (5.1%)
	I62V	4 (15.3%)
	I64LMV	4 (13.7%)
	G73CSTA	6 (8.1%)
	V77I	13 (4.6%)
	L89VIM	22 (3.6%)
	**L90M**	6 (16.2%)
**NRTI inhibitors**	**M41L**	2 (13.3%)
	A62V	2 (3.1%)
	**M184V**	2 (99.9%), 5 (82.8%), 9 (99.8%), 10 (100%), 16 (56.6%), 23 (68.5%)
**NNRTI inhibitors**	**K103N**	5 (100%), 10 (100%), 23 (99.8%)
	V108I	23 (59.8%)
	**E138KAGQR**	16 (7.6%)
	P225H	23 (55.3%), 10 (99.9%)
**Integrase inhibitors**	**S147G**	16 (7.6%)

Boldface font = major mutation. Regular font = accessory mutation.

**Table 4:** **Association between the major drug resistance mutations emerging in treatment failures from the ALIVE cohort, the drug regimens reported at the time of failure, and expected resistance profile based on genotype results**

**Sample # visit #**	**Drug resistance mutation**	**Gene product**	Frequency of DR mutation (coverage)*	**Mutational load (copies/ml)**	**Treatment at time point of failure**	**Stanford resistance profile for major mutations**
2v15	M41L	RT	13.3%(5316)	6913	ZDV, **3TC**	H: **3TC**, FTC
	**M184V**	RT	99.9%(7721)	51926		L: ABC, DDI
5v20	**K103N**	RT	100%(5807)	513564	ZDV, **3TC**,	H: **3TC**, FTC,
	**M184V**	RT	82.8%(5266)	425231	**ABC**, **EFV**	**EFV**, NVP
						L: **ABC**
						PL: DDI
6v20	*L90M*	PI	16.2%(3602)	179820	TDF, FTC, DRVr, RAL	H: NFV
						I: IDV, SQV
						L: ATV, LPV, FPV,
9v33	**M184V**	RT	99.8%(7493)	420158	ZDV, **3TC**, **ABC**, RTV, **FTC**	H: **3TC**, **FTC**
						L: **ABC**
						PL: DDI
10v10	**K103N**	RT	100%(5979)	40200	**EFV**, TDF, **FTC**	H: 3TC, **FTC**, **EFV**, NVP
	**M184V**	RT	100%(5399)	40200		L: ABC
						PL: DDI
16v11	*E138K*	RT	7.6%(8955)	27149	**3TC**, RTV, LPV	H: **3TC**, FTC
	**M184V**	RT	56.6%(8222)	202190		I: RPV
	*S147G*	IN	7.0%(3123)	25006		L: ABC
						PL: DDI, EFV, ETR, NVP
23v17	**K103N**	RT	99.8%(6110)	16168	**EFV**, TDF, **FTC**	H: 3TC, **FTC**, **EFV**, NVP
	**M184V**	RT	67.1%(6182)	10870		
						L: ABC
						PL: DDI
27v05	M46I**	PI	7.3%(3591)	1262	d4T, **3TC**, **ABC**, **IDVr**	L: NFV
						PL: ATV, FPV, **IDVr**, LPV (non-emerging DR related to treatment: H: **3TC**, L: **ABC**)

Boldface: correlation between mutation, treatment and drug resistance profile in each patient sample at the time point of treatment failure. H = high level drug resistance, I = intermediate drug resistance, L = low level drug resistance, PL: potential low level drug resistance. Drug resistance mutations in italics are not associated with any reported drug taken by the patients throughout the course of this study. *Coverage refers to the number of sequencing reads representing a given nucleotide. **Patient 27 virus also harbored I54V, L90M, K103N and M184V at both time points. These mutations did not emerge between treatment success and failure, but likely contributed to overall failure.

### Association of major drug resistance mutations with treatment in ALIVE

As a way to assess our drug resistance genotyping assay, we compared the major drug resistance mutations identified by our genotyping assay with the treatment regimens reported by the ALIVE study participants at the time of treatment failure. We define major drug resistance mutations as those included in the Stanford Drug Resistance Database matrices that are associated with high levels of phenotypic drug resistance [37,38]. This is opposed to accessory mutations whose role in treatment failure is less clear. Overall, 57% (8/14) of the patients with virus harboring any emerging drug resistance mutations relative to the first time point tested had major drug resistance mutations (Figure 3A and Table 3). We focused our mutation/treatment association analysis on these eight patients and analyzed the major mutations found in their virus using the Stanford drug resistance database Genotype Resistance Interpretation calculator [37,38]. The results of the calculator were then compared to each patient’s self-reported treatment regimen at the time of failure (Table 4). Of the eight patients harboring virus with major drug resistance mutations, seven patients had virus with mutations associated with a high level of drug resistance to one or more of the drugs in their regimen at the time point of failure (boldface in Table 4). Table 4 also shows the mutational load (viral load*frequency of mutation) of each mutation within each patient. M184V and K103N were the major mutations associated with treatment failure in this cohort and it is notable that the major drug resistance mutations that correlate with the drug regimen at the point of treatment failure are found at frequencies well above 50%, indicating drug selection pressure. As expected, the major mutations identified in most of the patients of this cohort using our genotyping technique associated well with the treatment regimens reported by the patients at the time of treatment failure.

Major drug resistance mutations emerging after treatment failure were then compared with all drugs self-reported for each individual during the course of all time points where data was collected. As shown in figure 3B, almost half (14/29) of all the 29 mutations emerging after treatment failure were major mutations. Overall, 10/14 major drug resistance mutations were associated with the patient self-reported drug regimens from either the time point of failure or previous time points (Table 4). These mutations were found at frequencies >50% while those that do not associate with the treatment regimen are found at <20%. Altogether, these data are consistent with the conclusion that this genotyping assay is detecting drug resistance mutations that would be expected based on the treatment regimens reported by the participants in the ALIVE cohort.

### Accessory drug resistance mutations detected in ALIVE

Accessory mutations are those that alone may not render resistance, but may contribute to resistance when combined with other mutations. All mutations identified on the International AIDS Society (IAS) USA drug resistance mutation list that were not part of the Stanford drug resistance database major mutation matrices were categorized as accessory mutations in this study. We identified accessory mutations in 23% of ALIVE patient samples that did not have major drug resistance mutations at the time of treatment failure (Figure 3A). Most of these mutations were present at frequencies <20% (Figure 3B and Table 3). While it is likely that these accessory mutations contributed to resistance, this cohort was not appropriately powered to explore the correlations between accessory mutations and drug resistance more definitively.

### Minority drug resistance variants in the ALIVE cohort

Currently, FDA-approved Sanger sequencing-based HIV drug resistance genotyping methods such as ViroSeq identify mutations present above a frequency of 20% within a host’s viral population [12,22]. Therefore, mutations found below this 20% frequency (i.e. minority variants) might be missed by current commercial techniques. As shown in figure 3B and detailed in Table 3, 59% (17/29) of all drug resistance mutations found in the ALIVE cohort by this genotyping technique were below the 20% frequency limit of detection of current genotyping. This indicates that the presented genotyping technique is likely more sensitive than those currently available commercially. Development of sensitive assays, like the genotyping assay presented here, will support future studies designed to delineate which minority mutations are important prior to the start of specific antiretroviral therapy. In our cohort, mutations likely contributing to treatment failure of a given drug regimen were predominantly present at frequencies >20%.

An important consideration when detecting lower frequency mutations or variants from samples with lower viral loads is reliability of the variant frequency detected and possibility of template resampling. While we were unable to do this with the presented cohort due to sample limitations, PCR amplifying a sample in triplicate and assessing the variant frequencies of each independent test for samples of different viral loads would be an important reliability test if adapting this methodology to clinical samples. In addition, when the number of sequences representing each nucleotide variant exceeds the number of virus genome templates (due to low viral loads) used to initiate the RT-PCR, then the sequences are representing each template more than once. This could result in false variant frequencies if there is a bias for one template over another during the PCR amplification. One way to address this concern would be to require higher viral loads or multiplex more samples on the sequencer to reduce the number of sequencing reads to below the number of templates added to the initial RT-PCR reaction. In this study, therefore, it is possible that some of the frequencies presented for samples with lower viral loads are not entirely accurate due to possible template resampling (Additional file 3). It remains questionable exactly what it means to have a mutation present at 10%, 50% or 99%. With our current understanding of drug resistance mutations, a mutation present at any frequency could quickly turn into a mutation present in nearly all viruses given drug selection pressure. Therefore, an important drug resistance mutation present at any frequency above the error rate would likely warrant recommendations for changes to drug regimens regardless of the actual frequency detected.

Lastly, one of the advantages of this MiSeq genotyping method includes detection of integrase inhibitor drug resistance mutations. One patient in the ALIVE cohort (patient 16) with no prior exposure to integrase inhibitors had a virus with a minority (7.6%) drug resistance mutation to Elvitegravir (Tables 3 and 4). Such a mutation should be considered when assigning a treatment regimen to this individual already failing their current antiretroviral regimen at the time of sample collection. The rollout of integrase inhibitors as part of initial treatment regimens increases the need for integrase inhibitor drug resistance testing prior to treatment initiation.

### Comparison of Illumina MiSeq platform with other platforms

We have already highlighted the advantages of the Illumina MiSeq platform over our previous work using the Roche/454 GS junior platform. Additionally, Roche plans to phase out 454 machines in 2016 making this platform obsolete for future development. This work is also conceptually similar to recent work from Gibson et al. describing comprehensive HIV drug resistance genotyping across multiple HIV group M subtypes for protease, reverse transcriptase and integrase inhibitors as well as coreceptor tropism using the Ion Torrent Personal Genome Machine [31]. There are, however, specific advantages of our methodologies using the MiSeq, including greater output (Illumina MiSeq: 13-15Gb vs. Ion Torrent PGM: 1Gb) and reduced systemic sequencing error rates (Illumina MiSeq: <0.4%, Ion Torrent: 1.78%) [25]. Fewer errors reduce analysis time and improve data reliability. In addition, our method is technically simpler, requiring a single fragmented PCR amplicon over the three required for the Gibson et al. method. Lastly, Illumina MiSeq is the first and only next-generation sequencing platform to receive FDA approval for assays developed on that device, making it the optimal deep sequencing platform to develop a HIV drug resistance assay for possible clinical use in the future [39].

### Data analysis considerations

Data analysis of next-generation sequencing techniques can be exceptionally difficult and requires many considerations. For example, when assessing viral variants related to HIV drug resistance one must consider the viral load of the samples to assess the possibility of template resampling and its effects on frequency determination, error associated with PCR amplification, systematic errors associated with sequencing, and accurate data analysis.

One of the advantages of the described data analysis approach is its relative simplicity using Geneious software. This software is very user-friendly and does not require a strong background in bioinformatics or programming skills. The parameters used in this analysis are fully defined in the Methods section and briefly include 3 basic steps: (1) pair the paired-end sequences together for each sample (2) trim ends based on quality and reference map against an annotated HXB2 sequence available here: (https://dholk.primate.wisc.edu/wiki/dho/public/page.view?name=default) (3) call variants relative to the HXB2 reference sequence. Steps 2 and 3 can be performed simultaneously for all samples in a sequencing run. Overall, only a few hours of hands-on time are necessary to complete steps 1, initiate step 2 and initiate step 3, with several hours of hands-off time to complete steps 2 and 3. Once the variants have been detected, the annotation table results can be exported out of Geneious and assessed in Microsoft Excel or in a text editor to determine which samples have which drug resistance mutations and their frequencies. Depending on the speed of the computer used to perform this analysis, a run of 64 samples could be fully analyzed within two days on a standard laptop with most of the analysis performed in Geneious being hands-off. A fully automated analysis approach would be required to use this method in a clinical setting, however, the provided methods are relatively quick and simple for research-based projects and is considerably faster than Sanger-based data analysis of 64 samples.

One of the sequence analysis challenges with working with HIV drug resistance is that resistance is defined by changes in amino acid sequence that could be rendered by several different nucleotide variants. Many analysis tools consider a single nucleotide variant at a time and not necessarily the effect of two nucleotide changes from a single sequence within a codon that might change an amino acid. For example, a variant caller might call a change from ATG (Met) to GTA (Val) as a mix of ATA (Ile) and GTG (Val), considering each single nucleotide change away from ATG as separate even though they are present in the same sequence. We have worked with the developers at Geneious to create a phased variant detection approach that is accurate when assessing nucleotide variants in the context of amino acid changes, which is paramount to our analysis approach. This variant detection approach is now built into the latest Geneious software release.

Lastly, while we can identify drug resistance mutations in the protease, reverse transcriptase and integrase regions simultaneously with this assay, given the nature of library formation and Illumina MiSeq sequencing we cannot necessarily associate together mutations found in one area of the pol gene product with mutations found in another area. With the paired-end technology it is possible to link one mutation from the same template to a mutation found ~600 bp downstream, but we cannot make those associations with mutations that span further apart. In this regard, this assay is not more informative than traditional genotyping assays using Sanger-based sequencing, which also cannot associate mutations together across multiple pol gene products. The only way to reliably span the entire pol gene from a single virus involves either cloning and sequencing or limiting dilution PCR and sequencing. However, sequencing technologies continue to improve and as error rates improve for techniques that sequence thousands of nucleotides in a single sequencing read, linking together all drug resistance mutations will become much easier.

## Conclusions

We have designed a universal primer set that amplifies part of the *pol* gene from the major HIV-1 subtypes A, B, C, D, CRF01_AE and CRF02_AG. These primers were used in an Illumina MiSeq-based sequencing method for the detection of drug resistance in primary HIV samples from individuals failing treatment after release from prison. We characterized drug resistance to protease, reverse transcriptase and integrase inhibitors simultaneously. 54% of the patients failing treatment harbored mutations in their virus at the time of treatment failure that were not present before treatment. Of the patients with virus harboring major drug resistance mutations, all but one patient had virus with mutations associated with resistance to the self-reported treatment regimen at the time of treatment failure. This finding suggests that virologic failure associated with incarceration may be due to the development of drug resistance mutations in about half of cases. This implies that actions to prevent drug resistance, such as preventing treatment interruption during and after incarceration, may reduce virologic failure associated with incarceration. 59% of all the mutations found were present at frequencies between 2% and 20% of the within-host virus population and would likely have been missed by traditional Sanger-based genotyping techniques. The technique presented in this manuscript should facilitate larger studies to elucidate the role(s) of these minority variants in treatment failure- an important consideration as genotyping moves to more sensitive sequencing platforms. Additionally, these methods can be adapted to sequence any region of the HIV virus by designing new primer sets to study a variety of questions related to HIV evolution. The results shown here suggest that the presented method is a significant advance over current HIV drug resistance genotyping by improving sensitivity, broadening the detection of mutations from protease through integrase, and improving genotyping in non-B HIV subtypes.

## Methods

### Ethics statement

The subjects used in this study provided informed written consent. The phlebotomy protocol and consent forms were approved by the Internal Review Board of Johns Hopkins University. The collected samples were de-identified and sent to the University of Wisconsin-Madison for sequencing. The protocol was approved by the Health Sciences Institutional Review Board at the University of Wisconsin-Madison (protocol number 2012–0140).

### Study subjects

Sixty-two participant samples were selected for inclusion in this study. These samples originated from individuals with a history of injection drug use who participated in the ALIVE study between 1997 and 2009 [29,40-42]. Selected participants were successfully treated with antiretroviral therapy (ART) prior to incarceration, but failed ART after release from prison. A baseline, pre-incarceration blood sample collected at a study visit before viral suppression was achieved and a follow-up sample collected after release from prison and treatment failure were obtained for this study (Additional file 3). As outlined in Additional file 3, many study participants were not drug naïve at the first time point we genotyped, but either failed their drug regimen or stopped taking antiretroviral drugs leading to an increase in viral load at the time point we sampled. Either a change in regimen or restarting a drug regimen resulted in viral suppression at the time point following the first time point we sampled. However, at a subsequent time point that coincided with the release from prison, individuals failed treatment again, which is the second time point that we tested. The range of viral load of these samples was 799–1,110,000 copies/ml. Treatment regimens were self-reported by the patients at each time point a sample was collected.

### HIV viral stocks

Viral stocks containing subtypes A, B, C, D, CRF01_AE, and CRF02_AG were obtained from the NIH AIDS Research and Reference Reagent Program, Division of AIDS, NIAID, NIH (cat. #11412) and used to test the subtype specificity of our primers. Viruses were provided by Robert Gallo, Smita Kularni, UNAIDS, Nelson Michael, and Victoria Polonis. The pHXBn-PLAP-IRES-N+ plasmid (cat. #3610) was obtained through the NIH AIDS research and reference reagent program, Division of AIDS, NIAID, NIH from Dr. Benjamin K. Chen and Dr. David Baltimore. To produce a clonal viral stock to assess error associated with our method, the pHXBnPLAP-IRES-N+ (HXBn) plasmid was transfected into 239 T packaging cells using the Xfect Transfection Reagent (Clontech). The supernatant was collected two days after transfection to limit virus production to a single round and the virus was quantitated using the Lenti-X qRT-PCR titration kit (Clontech). Viral RNA was isolated from this supernatant (viral load = 115,000 copies/ml) and subjected to one-step RT-PCR and sequenced using the same protocol that was used to sequence patient samples. The initial plasmid DNA was also sequenced using the same protocol as the research samples without the initial reverse transcription step. Both DNA and viral RNA starting templates are expected to have no variants relative to the HXB2 reference sequence (GenBank NC_001802). In addition, we sequenced the protease and reverse transcriptase gene products from the HIV plasmid used to generate the clonal virus using a Sanger-based approach as a standard to verify the pol gene sequence in the plasmid. This verification revealed two mutations in (HXB2 positions 1805 and 2473) the plasmid that were not expected based on the published map of the vector and GenBank entry. These mutations in the plasmid were found in 99% of the viral RNA sequenced from the clonal virus stock and were ignored during analysis as changes in the plasmid relative to the published HXB2 sequence. All other variants found by Illumina sequencing in either the plasmid or the viral stock were considered “false” variants that likely resulted from error in the PCR, sequencing, or analysis of the sequences. We used the maximum frequency found as “false” variants (0.9%) as the minimum frequency threshold for “real” variants found within a patient’s viral population (1.0%) that do not likely stem from error associated with the sequencing method.

### Primer design

Sequences representing multiple HIV subtypes were downloaded from the Los Alamos National Laboratory HIV database and aligned using the software program Geneious version 5.6. Regions of the *pol* gene flanking all known drug resistance mutations found in the protease, reverse transcriptase, and integrase protein coding regions were searched for conservation between group M HIV isolates. Two regions containing high conservation were identified and used to design PCR primers. Primers were optimized for melting temperatures, GC content and reduced hairpin and/or primer dimer formation as much as possible given restraints of the conserved regions (NetPrimer, Premier Biosoft International). The sequences of these primers named HIV2252-F and HIV5073-R can be found in Additional file 5 (nested primers). The forward primer contains a mismatch in the middle of the primer for some subtypes and is therefore designed with a degenerate nucleotide at that position (R = A/G). The reverse primer contained a mismatch in the middle of the sequence in some subtype C isolates. Two separate reverse primers were designed and tested with subtype C isolates. The primer that matched all group M isolates but contained a mismatch with some subtype C isolates amplified all subtypes well including all subtype C viruses tested and was used in this study (HIV5073-R). The expected PCR product size is 2892 bp. The external primers, as shown in Additional file 5, were previously designed (Todd Allen, personal communication).

### Sample preparation and PCR amplification

A schematic diagram representing the major steps to prepare samples for drug resistance genotyping using the method described in this paper is presented in Figure 1. Plasma from each sample was centrifuged at 14,000 x rpm at 4°C for 1 hour to concentrate virus for viral RNA extraction using the QIAamp Viral RNA Mini Kit (Qiagen). Viral RNA was subjected to one-step RT-PCR amplification using Superscript III Reverse Transcriptase and High Fidelity Platinum Taq Polymerase (Invitrogen) and the primers HIV2252-F and HIV5073-R. RT-PCR conditions were as follows: reverse transcription was performed at 50°C for 30 min., the enzyme was denatured at 94°C for 2 min., 40 cycles of amplification was performed at 94°C for 15 sec., 55°C for 30 sec., and 68°C for 3 min., followed by a 68°C extension for 10 minutes. Amplification was more reliable with the addition of 5% DMSO to the PCR reaction. Products were visualized on a DNA Flashgel (Lonza) and any sample containing PCR amplicons other than the product of interest was electrophoresed on a 1% agarose gel and the expected 2.8 kb amplicon was extracted using a Qiagen gel extraction MinElute kit (Qiagen). All samples were further purified using size exclusion beads (Agilent XP) to remove primer dimers at a 1:1 DNA:bead (volume) ratio. Samples that failed to amplify with a single round of PCR were subjected to nested PCR using the following primers in an external PCR (Additional file 5): HIV737-F and HIV5578-R. External PCR conditions included reverse transcription at 50°C for 60 min., polymerase activation at 94°C for 2 min., 2 cycles of 94°C for 15 sec., 60°C for 30 sec., and 68°C for 4 min. and 45 sec., 2 cycles of 94°C for 15 sec., 58°C for 30 sec., and 68°C for 4 min. and 45 sec., 31 cycles of 94°C for 15 sec., 55°C for 30 sec., and 68°C for 4 min. and 45 sec. followed by a 10 min. extension at 68°C. 5 μl of the 25 μl external PCR was used in a nested PCR using the conditions for single round PCR described above excluding the reverse transcription step. The nested primer set is shown in Additional file 5 and is the same primer set used for samples requiring only a single round of amplification.

### Amplicon fragmentation and sequencing

Following size exclusion purification, PCR amplicons were quantitated using picogreen dye and the Qubit (Invitrogen) fluorometer or DTX 800 Multimode detector plate reader (Beckman Coulter). 1 ng of each sample was fragmented using Nextera XT transposon technology (Epicentre). The average size of the fragmented PCR amplicons was 774 bp (range 519-1060 bp) as detected by bioanalysis (Agilent). Following Nextera tagmentation, Illumina sequencing adaptors and molecular tags were added via PCR, size exclusion bead purification was performed, and samples were quantitated using picogreen as previously described. Individual samples were then pooled at equimolar ratios (1–2 nM/sample, depending on run) based on the mean size of each library. The final diluted 8–9 pM pool was denatured using sodium hydroxide according to the Illumina MiSeq protocol and 1% denatured PhiX beads were added as a sequencing control. Samples were loaded into a 300-cycle MiSeq cartridge and sequenced This protocol is also amenable to the updated Illumina 500 and 600 cycle MiSeq kits.

### Data analysis

Data was analyzed using Geneious version 5.6 software. A HXB2 reference sequence (GenBank accession # NC_001802) was uploaded into Geneious and all drug resistance mutations were manually annotated onto the sequence to generate an annotated reference sequence for reference-based assembly (https://dholk.primate.wisc.edu/wiki/dho/public/page.view?name=default). Sequences are demultiplexed automatically on the MiSeq as part of the data processing steps and two paired .fastq files are generated for each sample representing the two paired-end reads. After importing the .fastq files from the MiSeq into Geneious, the two sequence lists from each sample were paired. Next, all sequences were trimmed at the ends as part of the assembly process using the modified-Mott algorithm and quality scores assigned by the sequencing base caller. This algorithm trims the ends to the point where trimming no longer improves the error rate by more than the error probability limit threshold set, in this case 0.001, or a 0.1% error rate. Sequences were then mapped to the annotated reference sequence with the following parameters: Gaps were allowed with a maximum of 15% per read and gap size of 15, word length was set to 14, maximum mismatches per read were set to 25%, minimum overlap identity was set to 80%, maximum ambiguity was set to 16 and “search more thoroughly for poor matching reads” was selected. In general, the reference assembler uses a seed and expand-type mapper, followed by a fine-tuning step that was set to none (fast / read mapping) for this analysis. All nucleotide variants represented by at least 5 sequencing reads and at a frequency >1% from the reference sequence were then called using the variant finder. This threshold was set based on previous work to establish minimum sequencing read coverage for next-generation sequencing [22,30]. To ensure that variants were called relative to the correct codon, the “merge adjacent variations” and “use separate annotations for each variant at a position” were selected. Geneious developed a variant finder analysis tool that allows amino acid variants within a codon to be called together when present on the same sequence to accurately reflect the effects of all single nucleotide changes on the amino acid sequence. This variant finder was used as a plugin with Geneious 5.6 at the time of this analysis but has since been incorporated into Geneious starting with version 6.1 and is now part of their standard Geneious Pro software. Variants and their frequencies were exported into an excel document and filtered for those present in amino acid sites known to correlate with drug resistance based on the Stanford drug resistance database list and the 2012 list of IAS-USA HIV drug resistance mutations as annotated on the reference sequence. Frequency of each variant and the number of sequences representing each nucleotide position containing a variant away from the reference sequence was also calculated by the variant finder plugin.

### Phylogenetic analysis

A consensus sequence was constructed from the reference-based assemblies performed on each sample using the 50% strict setting. These nucleotide sequences were aligned by the CLUSTAL algorithm in MEGA 6 [43]. A maximum likelihood (ML) phylogenetic analysis was conducted based on the GTR + G + I model, which was chosen using the Bayes Information Criterion in MEGA 6. The reliability of the clustering patterns in ML trees was tested by bootstrapping; 1000 bootstrap pseudo-samples were used.

## Additional files

Additional file 1:
**1.0% agarose gel image of RT-PCR products amplified with universal primers from 19 NIH HIV isolates.**
Additional file 2:
**All non-synonymous variants detected while sequencing HXB2 clonal stock viruses from three independent PCR amplifications and two independent sequencing reactions to determine the error rates associated with this sequencing method.**
Additional file 3:
**Patient information from samples collected in the ALIVE cohort.**
Additional file 4:
**Maximum likelihood phylogenetic analysis of the assembled sequences from the ALIVE cohort with reference sequences representing subtypes A, B, C, D, CRF01_AE and CRF02_AG using the Bayes Information Criterion in MEGA6.**
Additional file 5:
**External and nested primers used to RT-PCR amplify HIV.**
