# Supplementary material for: Cross-clade simultaneous HIV drug resistance genotyping for reverse transcriptase, protease, and integrase inhibitor mutations by Illumina MiSeq
Source: Retrovirology. 2014 Dec 23;11:122. doi: 10.1186/s12977-014-0122-8 (PMC4302432; doi:10.1186/s12977-014-0122-8)

Additional file 1: 1.0% agarose gel image of RT-PCR products amplified with universal primers from 19 NIH HIV isolates.

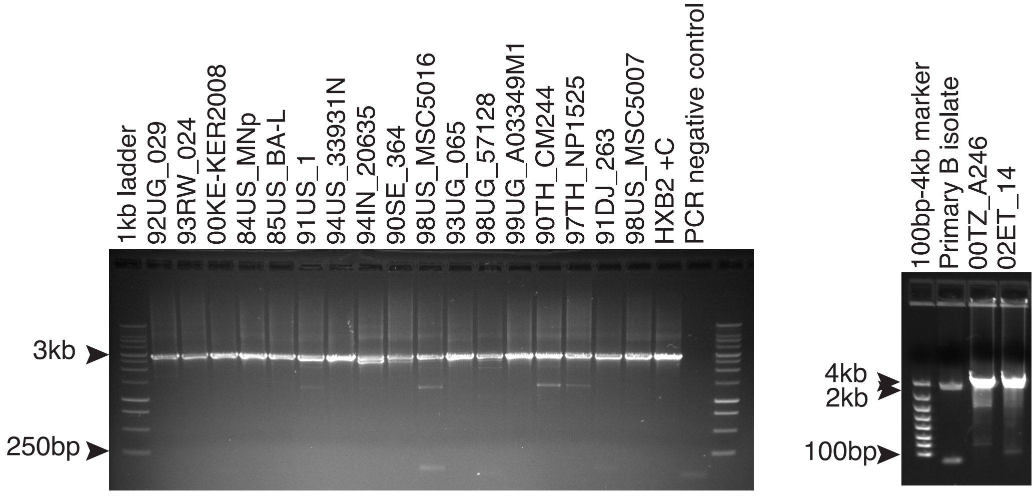

Supplement: Additional file 1: — 1.0% agarose gel image of RT-PCR products amplified with universal primers from 19 NIH HIV isolates. [file 12977_2014_122_MOESM1_ESM.pdf]
