# Supplementary material for: Cross-clade simultaneous HIV drug resistance genotyping for reverse transcriptase, protease, and integrase inhibitor mutations by Illumina MiSeq
Source: Retrovirology. 2014 Dec 23;11:122. doi: 10.1186/s12977-014-0122-8 (PMC4302432; doi:10.1186/s12977-014-0122-8)
Supplement: Additional file 3: — Patient information from samples collected in the ALIVE cohort. [file 12977_2014_122_MOESM3_ESM.pdf]

Additional file 3: Patient information from samples collected in the ALIVE cohort.

Patient #	Visit	Before treatment/ after failure	Visit date	Viral load (copies/ml)	Single/ Nested PCR	Sequenced (Yes/No)	Drug naïve before first visit?
**1**	v04	Before	7/99	38737	**Nested**	Y	No
	v06	After	4/04	41855	Single	Y	
	v12	After	7/07	133000	Single	Y	
**2**	v13	Before	6/98	19244	Single	Y	No
	v15	After	12/99	51978	Single	Y	
**3**	v22	Before	9/03	157397	Single	Y	No
	v24	After	3/06	2550	Single	Y	
**4**	v22	Before	8/98	3132	Single	Y	No
	v24	After	9/99	1070	Single	Y	
**5**	v18	Before	12/00	305059	Single	Y	No
	v20	After	7/02	513564	Single	Y	
**6**	v18	Before	4/08	281500	Single	Y	No
	v20	After	5/09	1110000	Single	Y	
**7**	v14	Before	8/99	265878	Single	Y	No
	v16	After	8/00	337712	Single	Y	
**8**	v03	Before	9/98	51852	Single	Y	No
	v06	After	6/00	944	**Did not amplify**	N	
**9**	v30	Before	5/07	421000	Single	Y	No
	v32	After	11/08	799	**Nested**	Y	
	v33	After	5/09	22600	Single	Y	
**10**	v07	Before	12/98	3721	Single	Y	No
	v10	After	7/08	40200	Single	Y	
**11**	v19	Before	5/98	21402	Single	Y	No
	v21	After	4/99	16988	Single	Y	
**12**	v21	Before	4/99	37804	**Did not amplify**	N	No
	v25	After	5/01	4231	Single	Y	
**13**	v01	Before	7/98	30172	Single	Y	Yes
	v04	After	5/00	32789	Single	Y	
**14**	v19	Before	2/99	20558	Single	Y	No
	v21	After	6/00	118870	Single	Y	
**15**	v16	Before	3/05	3200	Single	Y	No
	v18	After	12/06	480000	Single	Y	
**16**	v09	Before	4/03	357227	Single	Y	No
	v11	After	3/04	43535	Single	Y	
**17**	v11	Before	8/07	28900	Single	Y	No
	v13	After	10/08	66200	Single	Y	
**18**	s01	Before	3/06	69700	Single	Y	Yes
	v06	After	8/09	41000	Single	Y	
**19**	v02	Before	11/07	3750	**Nested**	Y	Yes
	v04	After	10/08	206000	Single	Y	
**20**	v08	Before	12/03	1604	**Nested**	Y	Yes
	v10	After	2/06	934	**Nested**	Y	
**21**	v04	Before	9/97	11264	Single	Y	No

	v12	After	8/04	1166	**Nested**	Y	
**22**	v08	Before	8/99	103077	Single	Y	Yes
	v10	After	2/01	6869	Single	Y	
**23**	v04	Before	10/99	5689	Single	Y	No
	v07	After	4/03	66260	Single	Y	
	v15	After	3/07	1190	**Nested-*sequence failed**	N	
	v17	After	1/09	16200	Single	Y	
**24**	v03	Before	12/99	17206	Single	Y	No
	v05	After	2/01	5540	Single	Y	
**25**	v11	Before	12/05	35000	Single	Y	No
	v14	After	6/07	25200	Single	Y	
**26**	v04	Before	11/99	187807	Single	Y	No
	v06	After	12/00	456974	Single	Y	
**27**	v02	Before	3/99	17281	**Nested**	Y	Yes
	v05	After	9/00	8100	Single	Y	
**28**	v12	Before	8/04	4345	**Nested-*sequence failed**	N	No
	v14	After	8/05	181000	Single	Y	
**29**	v05	Before	8/08	1350	**Nested**	Y	No
	v07	After	10/09	909	**Nested**	Y	
