# Supplementary material for: Cross-clade simultaneous HIV drug resistance genotyping for reverse transcriptase, protease, and integrase inhibitor mutations by Illumina MiSeq
Source: Retrovirology. 2014 Dec 23;11:122. doi: 10.1186/s12977-014-0122-8 (PMC4302432; doi:10.1186/s12977-014-0122-8)
Supplement: Additional file 5: — External and nested primers used to RT-PCR amplify HIV. [file 12977_2014_122_MOESM5_ESM.pdf]

Additional file 5: External and nested primers used to RT-PCR amplify HIV.

Primer description	Primer name	Primer sequence
External Forward	HIV737-F	5'-GCG RCT GGT GAG TAC GCC
External Reverse	HIV5578-R	5'-TCT TCY GGG GCT TGT TCC
Nested Forward	HIV2252-F	5'-CCC TCA RAT CAC TCT TTG GC
Nested Reverse	HIV5073-R	5'-CCA CAC AAT CAT CAC CTG CC
