# Supplementary material for: Cross-clade simultaneous HIV drug resistance genotyping for reverse transcriptase, protease, and integrase inhibitor mutations by Illumina MiSeq
Source: Retrovirology. 2014 Dec 23;11:122. doi: 10.1186/s12977-014-0122-8 (PMC4302432; doi:10.1186/s12977-014-0122-8)
Supplement: Additional file 4: — Maximum likelihood phylogenetic analysis of the assembled sequences from the ALIVE cohort with reference sequences representing subtypes A, B, C, D, CRF01_AE and CRF02_AG using the Bayes Information Criterion in MEGA6. [file 12977_2014_122_MOESM4_ESM.pdf]

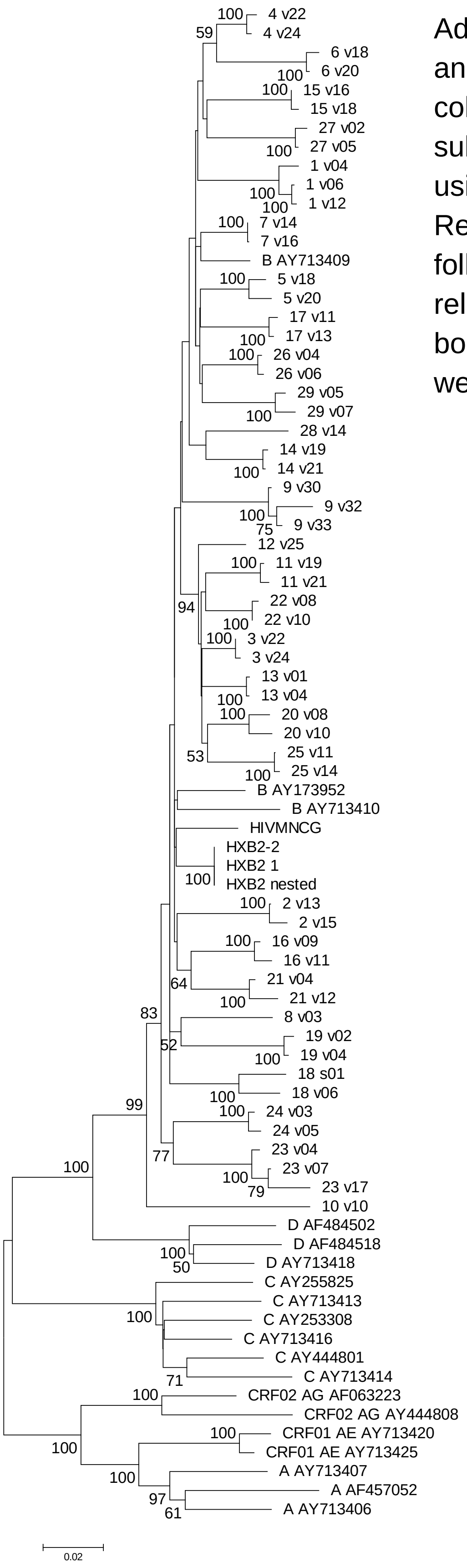

Additional file 4: Maximum likelihood phylogenetic analysis of the assembled sequences from the ALIVE cohort with reference sequences representing subtypes A, B, C, D, CRF01_AE and CRF02_AG using the Bayes Information Criterion in MEGA6. Reference sequences are indicated by the subtype following by the Genbank accession number. The reliability of the clustering patterns were tested by bootstrapping and 1000 bootstrap pseudo-samples were used.
